# The Transcription Factor ATF4 Promotes Expression of Cell Stress Genes and Cardiomyocyte Death in a Cellular Model of Atrial Fibrillation

**DOI:** 10.1155/2018/3694362

**Published:** 2018-05-29

**Authors:** Johanna K. Freundt, Gerrit Frommeyer, Fabian Wötzel, Andreas Huge, Andreas Hoffmeier, Sven Martens, Lars Eckardt, Philipp S. Lange

**Affiliations:** ^1^Department of Cardiovascular Medicine, Division of Electrophysiology, University Hospital Münster, Germany; ^2^Institute of Physiology II, University of Münster, Germany; ^3^Department of Pathology, University Hospital Münster, Germany; ^4^Core Facility Genomik, Medical Faculty, Westfälische Wilhelms-Universität Münster, Germany; ^5^Department of Cardiac and Thoracic Surgical, University Hospital Münster, Germany

## Abstract

**Introduction:**

Cardiomyocyte remodelling in atrial fibrillation (AF) has been associated with both oxidative stress and endoplasmic reticulum (ER) stress and is accompanied by a complex transcriptional regulation. Here, we investigated the role the oxidative stress and ER stress responsive bZIP transcription factor ATF4 plays in atrial cardiomyocyte viability and AF induced gene expression.

**Methods:**

HL-1 cardiomyocytes were subjected to rapid field stimulation. Forced expression of ATF4 was achieved by adenoviral gene transfer. Using global gene expression analysis and chromatin immunoprecipitation, ATF4 dependent transcriptional regulation was studied, and tissue specimen of AF patients was analysed by immunohistochemistry.

**Results:**

Oxidative stress and ER stress caused a significant reduction in cardiomyocyte viability and were associated with an induction of ATF4. Accordingly, ATF4 was also induced by rapid field stimulation mimicking AF. Forced expression of wild type ATF4 promoted cardiomyocyte death. ATF4 was demonstrated to bind to the promoters of several cell stress genes and to induce the expression of a number of ATF4 dependent stress responsive genes. Moreover, immunohistochemical analyses showed that ATF4 is expressed in the nuclei of cardiomyocytes of tissue specimen obtained from AF patients.

**Conclusion:**

ATF4 is expressed in human atrial cardiomyocytes and is induced in response to different types of cell stress. High rate electrical field stimulation seems to result in ATF4 induction, and forced expression of ATF4 reduces cardiomyocyte viability.

## 1. Introduction

Atrial fibrillation (AF) is the most common arrhythmia in industrialized countries with an increasing burden of morbidity. However, despite the overwhelming clinical relevance of AF, fundamental mechanisms governing the maintenance and perpetuation of atrial fibrillation remain poorly understood. Known pathophysiological mechanisms include but are not limited to oxidative stress, abnormal Ca^2+^ homeostasis, ion channel dysfunction, and microRNA mediated dysregulation [1]. However, key molecular and electrophysiological changes leading to AF and to disease progression have not yet been fully elucidated. As a consequence of our limited understanding of the complex pathophysiology of AF, the prevention and treatment strategies of this arrhythmia still need to be optimized.

Atrial remodeling is characterized by complex structural and electrical changes leading to atrial dilatation and atrial fibrosis thereby promoting conduction slowing, spontaneous depolarizations and action-potential duration prolongation. Cardiomyocyte apoptosis is assumed to play an important role in atrial remodeling and disease progression [2, 3]. In fact, the ageing heart is experiencing a constant loss of cardiomyocyte (estimated 0.5–1% per year [1, 4]), and fibrous tissue often replaces cardiomyocytes undergoing cell death. Moreover, age is one of the major risk factors for the development of AF [4].

Oxidative stress, calcium overload, and endoplasmic reticulum (ER) stress seem to play important roles in AF and AF induced atrial remodeling [5, 6]. In many cells, including cardiomyocytes, the expression of genes that can mitigate the consequences of oxidative stress and ER stress is precisely coordinated by a synergistic network of stress-sensing signaling cascades [7–9]. Specifically, ER stress caused by calcium overload and other stressors and aberrantly elevated levels of oxidants can trigger the transcriptional induction of a number of adaptive pathways [10]. This cellular stress response is tightly controlled by a family of stress-responsive transcription factors. Among these transcription factors, the activating transcription factor 4 (ATF4)/cAMP response element binding protein 2 might be particularly important. While being constitutively expressed only at low concentrations, ATF4 can be rapidly induced under particular cell-stress conditions [11]. ER stress leads to decreased translation of most cellular mRNAs; paradoxically, the mRNA that encodes activator of ATF4 is translated more efficiently. Increased levels of ATF4 serve important roles as a transcriptional inducer of a certain ER stress response genes, which assist in recovery from the stress [12]. During the prosurvival phase of the ER stress response ATF4 induces numerous genes involved in resolution of the ER stress, such as genes that encode amino acid transporters and ER resident chaperones [13]. However, after prolonged ER stress, continued ATF4 expression mediates the upregulation of genes that contribute to programmed cell death. For example, ATF4 induces the transcription factor C/EBP homologous protein [14, 15], which induces numerous proapoptotic proteins, including GADD34 [16], and Tribbles-related protein 3 [17]. Moreover, CHOP regulates expression of several Bcl2 family members [15, 18, 19]. ATF4 binds to the promoter regions of several different target genes, including many involved in ER stress and redox control. In fibroblasts, ATF4 has an important role in the cellular response to amino acid depletion, oxidative stress, and endoplasmic reticulum stress and helps to balance redox homeostasis. More specifically, ATF4-deficient fibroblasts have been shown to be prone to death when exposed to different types of stresses, including oxidative stress and amino acid deprivation [20]. To get a more conclusive insight in the mechanism of AF-induced myocyte remodeling, we used a cell culture based model of atrial fibrillation. Rapid pacing of cultured atrial-derived myocytes HL-1 mimics the phenotypic feature of tachycardia-induced atrial cardiomyocyte remodelling* in vivo* [21, 22]. Here, we show that ATF4 expression impairs atrial cardiomyocyte survival in an* in vitro* model of atrial fibrillation. Moreover, we demonstrate that ATF4 is expressed at relevant levels in atrial cardiomyocytes* in vivo* and might play an important role in atrial remodeling in response to atrial fibrillation.

## 2. Materials and Methods

A detailed description of the methods can be found in the supplements.

### 2.1. Immunohistochemistry

Atrial appendage tissue was obtained from 17 individual patients undergoing cardiac surgery with (*n* = 9) and without atrial fibrillation (*n* = 9). Patient characteristics are described in Table 2. The study was approved by the University of Münster Ethical Committee and Institutional Review Board. Therefore, it has been performed in accordance with the ethical standards laid down in the 1964 Declaration of Helsinki and its later amendments. All patients gave their written informed consent for the histological examination. ATF4 was detected with a commercially available antibody (rabbit polyclonal anti-ATF4, 1 : 200, LS-B3517, LSBio). ATF4 positive nuclei were counted in representative fields of all patient specimen. mRNA was extracted and subjected to real-time PCR.

### 2.2. HL-1 Cell Culture and Pacing

The murine cardiomyocyte cell line HL-1 was kindly provided by Dr. Claycomb, Lousiana State University [23, 24]. The cell line was cultured in Claycomb medium (Sigma) supplemented with 0.1 mM norepinephrine (Sigma), 2 mM L-glutamine (Biochrom), 100 U/mL penicillin (Biochrom), 100 *μ*g/mL streptomycin (Biochrom), and 10% fetal bovine serum (Sigma). The myocytes were cultured in flasks coated with 5 *μ*g/ml fibronectin (Sigma) and 0.02% gelatin (Sigma), in a 5% CO_2_ atmosphere at 37°C. They were split at full confluence with 0.05%/0.02% trypsin-EDTA (Biochrom) 1 : 2 or 1 : 3 and media were changed every 24 to 48 hours. Rapid pacing of HL-1 cells was performed with the C-Pace EP Culture Stimulation System (IonOptix). To induce tachycardia, HL-1 myocytes were cultured on coverslips in 35 mm dishes. At 80% confluence the coverslips were embedded into the C-Dish between the carbon electrodes and fresh medium was added. After 1 h at 37°C the cells were subjected to an electrical field stimulation (1 Hz, 10 ms, 20 V, biphasic waveform) for 1 h and then to a modified electrical field stimulation (4 Hz, 10 ms, 20 V) for 20 h. 1 *μ*M Thapsigargin was added to the HL-1 medium and incubated for 18 h. 0–0.1 *μ*l/ml H_2_O_2_ was incubated for 6 h. 2 and 4 *μ*g/ml tunicamycin were added to the HL-1 medium and incubated for 5 h.

### 2.3. Isolation of Cardiac Fibroblasts

The cardiac fibroblasts were isolated from ventricles from adult C57BL/6 mice.

### 2.4. Infection of HL-1 Cells and Fibroblasts with Adenovirus

Cells were infected with virus particles containing GFP (vVQ-pEF-GFP-mycTag-K-NpA), ATF4 wild type (vVQ-pEF-ATF4wt-mycTag-K-NpA), or ATF4ΔRK (vVQ-pEF-ATF4ΔRK-mycTag-K-NpA) (ViraQuest Inc.) as described elsewhere [25].

### 2.5. Preparations of Protein Lysates and Western Blot Analyses

Western blot membranes were incubated with the following primary antibodies: anti-ß-actin (sc-1616, 1 : 1000, Santa Cruz) and anti-ATF4 (sc-200x, 1 : 5000, Santa Cruz) and secondary antibody anti-goat (1 : 5000, Santa Cruz) and anti-rabbit (1 : 1000, Dako). Complete western blots are shown in Supplemental Figure S2.

### 2.6. Quantitative Real-Time PCR Analysis

Real-time PCR analysis with cDNA from HL-1 cells was performed using TaqMan Gene Expression Master Mix (Applied Biosystems) with the following primers: ATF4 (Mm00515325_g1, Applied Biosystems) and ß-actin (Mm00607939_s1, Applied Biosystems). Real-time PCR analysis from human tissue was performed using Sybr Green qPCR Master Mix (Roboklon) with the following primers: ATF4 (forward primer: TCAAACCTCATGGGTTCTCC, reverse primer: GTGTCATCCAACGTGGTCAG) and ß-actin (forward primer: ATTGCCGACAGGATGCAGAA, reverse primer: ACATCTGCTGGAAGGTGGACAG).

### 2.7. Microarray Analysis

HL-1 cells were infected with Adenovirus containing a GFP, ATF4wt, or ATF4ΔRK construct. After 48 h, cells were rapidly paced for 20 h. Biotin-labeled cRNA was hybridized on Illumina MouseWG-6 v2.0 expression BeadChips. In the Illumina GenomeStudio Software 2011.1, raw data were normalized using the quantile algorithm. Differential gene expression was assessed on the basis of grouped replicates and thresholds for expression ratios or, alternatively, for both expression ratios and statistical significance employing the *t*-test model, based on standard deviations between biological replicates. Filtering of genes was performed using sorting and autofiltering functions in MS Excel (*p* value ≤ 0.05). Functional annotation analysis was carried out using DAVID 6.7.

### 2.8. Chromatin Immunoprecipitation (ChIP) Assay

HL-1 cells were infected with Adenovirus containing a GFP, ATF4wt, or ATF4ΔRK construct. DNA binding proteins were cross-linked for 10 min in 0.5% formaldehyde. The HL-1 cells were lysed and sonicated at 40% amplitude for 10 s on, 20 s off, and 20 cycles on ice. Each sample was incubated with anti-myc antibody (9B11, Cell Signalling) overnight at 4°C. The ChlP-DNA was added to a library preparation (NEBNext ChIP Seq Library Preparation), and a single read sequencing was performed (Core Facility Genomik University of Münster) using the NextSeq 500 System. The algorithm MACS2 was used for identifying transcript factor binding sites. Functional annotation analysis was carried out using DAVID 6.7. The software package BETA was used to compare genes from the ChIP Assay and the Microarray analysis. Filtering of genes was performed using sorting and autofiltering functions in MS Excel (fold change ≥ 1.2 and ≤0.83).

### 2.9. Statistical Analysis

Two-sample independent Student's *t*-tests were used to compare the means of two groups (SPSS Version 22, SPSS). Differences with a *p* value of ≤0.05 were considered to be statistically significant.

## 3. Results

**Figure 1:**
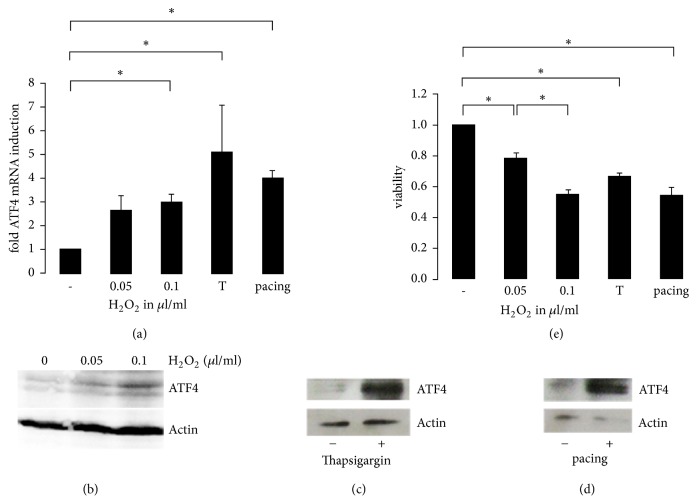
*ATF4 expression in H*_2_ *O*_2_ *and thapsigargin treated cardiomyocytes and in electrically stimulated cardiomyocytes*. (a) Real-time PCR of ATF4 mRNA expression in cultured HL-1 cardiomyocytes in response to treatment with 0, 0.05, or 0.1 *μ*l/ml H_2_O_2_ (*n* = 3), real-time PCR of ATF4 mRNA expression in cultured cardiomyocytes in response to treatment with 1 *μ*M thapsigargin (*n* = 3), and real-time PCR of cardiomyocytes stimulated electrically with 4 Hz for 20 h (*n* = 3). Representative western blots showing protein expression of ATF4 in response to H_2_O_2_ treatment (b) and in response to thapsigargin treatment (c). (d) Cardiomyocytes were electrically stimulated with 4 Hz for 20 h and ATF4 protein expression was detected. (e) MTT assay displaying viability of cardiomyocytes treated with H_2_O_2_ (*n* = 3), 1 *μ*M thapsigargin (*n* = 3), and paced HL-1 cardiomyocytes (*n* = 5). *∗* indicates *p* < 0.05.

Both oxidative stress and ER stress play key roles in the atrial remodeling process in atrial fibrillation. ATF4 is a key transcription factor mediating cellular gene expression changes in response to different types of cell stress. Therefore, we initially hypothesized that ATF4 could be induced by both oxidative stress and ER stress in atrial cardiomyocytes. We initiated our study with an analysis of ATF4 expression on mRNA and protein level in the atrial cardiomyocyte cell line HL-1 in response to both oxidative stress and ER stress. We analyzed the expression of ATF4 in response to oxidative stress induced by treatment with hydrogen peroxide. Accordingly, oxidative stress heightened ATF4 expression level on both the mRNA and protein level (Figures 1(a) and 1(b)). ER stress in atrial cardiomyocytes was induced by thapsigargin, an agent that raises the cytosolic calcium concentration thereby mimicking calcium overload, a condition that has been associated with endoplasmic reticulum stress. Treatment of cardiomyocytes with thapsigargin caused an increase of ATF4 mRNA and ATF4 protein expression (Figures 1(a) and 1(c)). Correspondingly, treatment with tunicamycin, a well characterized ER stress inducing agent, also led to an increased level of ATF4 expression (Supplemental Figure 1). Both oxidative stress and ER stress were associated with a decrease in cell viability as measured by MTT assay (Figure 1(e)). Thus, we hypothesized that ATF4 expression might also be elevated in response to atrial fibrillation, a condition that has been associated both with oxidative stress and ER stress. In order to study the response to atrial fibrillation in atrial cardiomyocytes, we used an established cellular model of AF, in which HL-1 cardiomyocytes are subjected to rapid field stimulation. Accordingly, rapid field stimulation led to an induction of ATF4 mRNA, an increased ATF4 expression on the protein level, and decreased cell viability (Figures 1(a), 1(d), and 1(e)).

**Figure 2:**
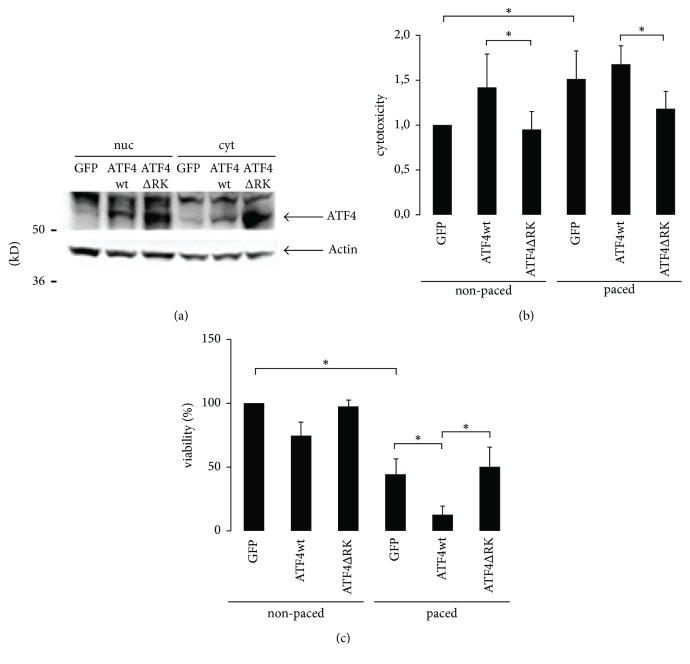
*Effect of pacing on viability in cardiomyocytes overexpressing ATF4*. (a) Representative western blots showing nuclear (nuc) and cytosolic (cyt) protein expression of ATF4 in cardiomyocytes overexpressing GFP, ATF4wt, or ATF4ΔRK. GFP, ATF4wt, or ATF4ΔRK were overexpressed in cardiomyocytes followed by pacing. Cytotoxicity was measured by LDH-assay (*n* = 3) (b) and viability was measured by MTT-Assay (*n* = 4) (c), *∗* indicates *p* < 0.05.

To further investigate the role of ATF4 in cardiomyocytes, we continued our study analyzing the effect of forced expression of ATF4 in atrial cardiomyocytes. Adenoviral vectors were used to overexpress wild type ATF4 in HL-1 cells. For a negative control, we used GFP or a dominant negative ATF4 mutant (ATF4ΔRK) (Figure 2(a)). In general, cytotoxicity was raised by electrical stimulation of HL-1 cells (Figure 2(b)). Forced expression of ATF4 further increased cytotoxicity in electrically stimulated HL-1 cardiomyocytes compared to HL-1 cells infected with GFP or with ATF4ΔRK. Accordingly, an MTT assay showed a reduced viability of the electrically stimulated cells and a further reduction of viability by expression of wild type ATF4 (Figure 2(c)).

**Figure 3:**
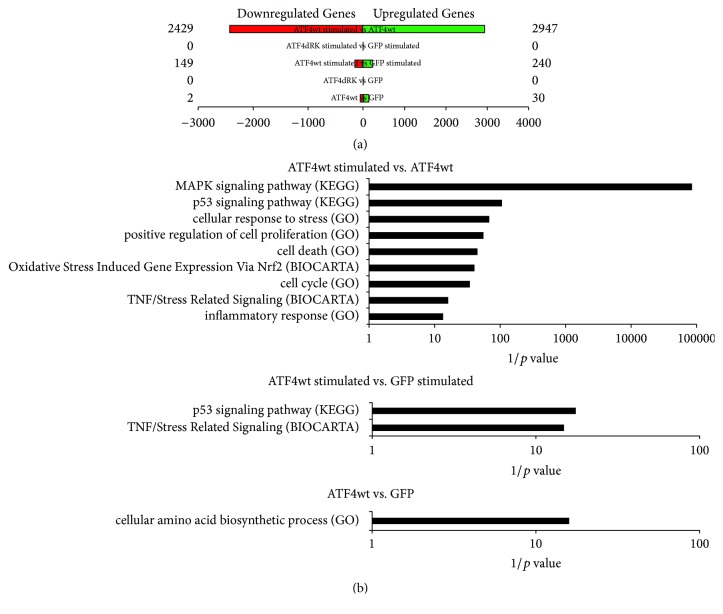
*Gene expression microarray analysis*. (a) The number of genes compared to control (GFP) that are up- (green) and downregulated (red) in cardiomyocytes overexpressing ATF4 wild type (wt) or ATF4ΔRK with and without electrical stimulation (fold change ≥ 1.2 or ≤0.83, and *p* value ≤ 0.05). (b) Pathways enriched in electrically stimulated ATF4wt overexpressing HL-1 cardiomyocytes versus unstimulated ATF4wt overexpressing HL-1 cardiomyocytes (upper panel), electrically stimulated ATF4wt overexpressing cardiomyocytes versus stimulated GFP overexpressing cardiomyocytes (middle panel), and ATF4wt versus GFP overexpressing cardiomyocytes (lower panel).

In order to decipher the gene expression changes associated with electrical stimulation and ATF4 expression, a global analysis of ATF4 target genes was carried out by overexpression of ATF4, ATF4ΔRK, or GFP in electrically stimulated and nonstimulated HL-1 cardiomyocytes and subsequent microarray analysis. In nonstimulated cardiomyocytes, overexpression of ATF4 led only to a minor change in gene expression (Figure 3(a)). 30 genes were upregulated and 2 genes were downregulated by ATF4 compared to cells infected with GFP-vector. The ATF4 mutant did not display regulatory properties in nonstimulated cardiomyocytes (comparison of ATF4ΔRK versus GFP overexpressing cardiomyocytes). However, in electrically stimulated cardiomyocytes, ATF4 caused a relevant change in gene expression (comparison of ATF4wt stimulated versus GFP stimulated). 240 genes were upregulated and 149 genes were downregulated as a consequence of ATF4 overexpression in stimulated cells compared to electrically stimulated cells with GFP overexpression. The most profound gene expression differences were observed when comparing ATF4 overexpressing, electrically unstimulated cardiomyocytes to ATF4 overexpressing, and electrically stimulated cardiomyocytes. Electrical stimulation in ATF4 overexpressing cardiomyocytes caused an upregulation of 2947 genes and a downregulation of 2429 genes (Figure 3(a) and Table 1(a)). Pathway enrichment analysis in this dataset (Figure 3(b), upper panel) revealed that electrical stimulation in ATF4 overexpressing cardiomyocytes led to a pattern of gene expression characteristic of the cellular response to ER stress, oxidative stress, inflammation, and cell death including the upregulation of genes that are involved in the MAPK signaling pathway. ATF3, Dusp2, and Fos belong to this group of genes (Table 1(a)). ATF3 is a member of the mammalian activation transcription factor/cAMP responsive element-binding (CREB) protein family of transcription factors and is known to be induced in response to different types of cell stress; Dusp2 and Fos are genes of the MAPK signaling pathway.

In electrically stimulated cells, ATF4 overexpression led to a regulation of the p53 signaling pathways and TNF/stress related signaling (Figure 3(b), middle panel). On the contrary, ATF4 overexpression in electrically unstimulated HL-1 cardiomyocytes mainly influenced the expression of genes involved in amino acid biosynthesis (Figure 3(b), lower panel).

**Figure 4:**
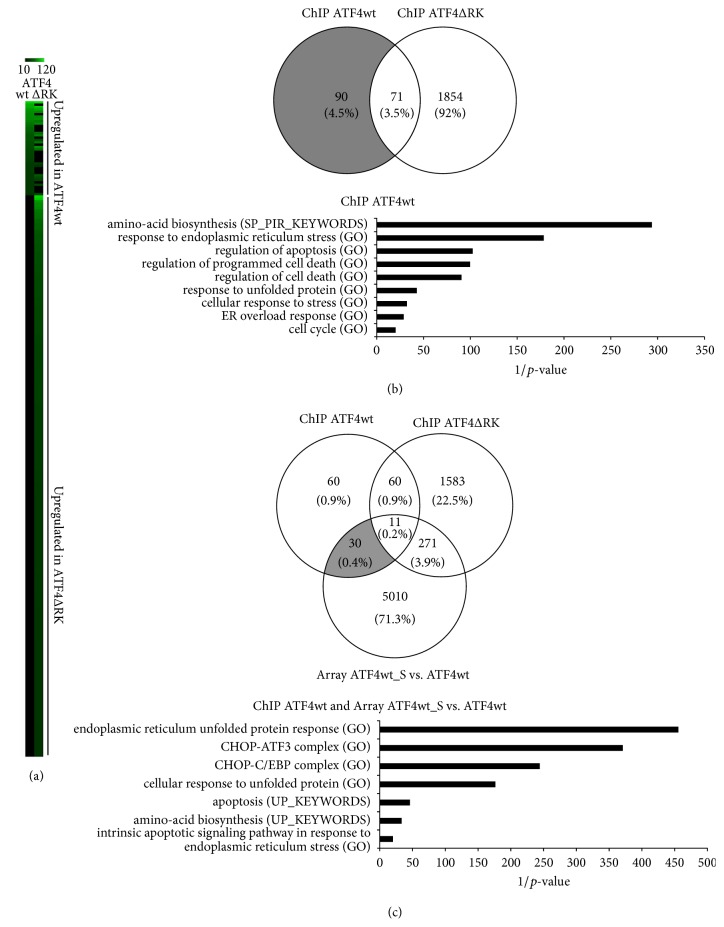
*ChIP-on-chip Assay*. (a) Heatmap representation comparing genes with a –log⁡*p* value > 10 expressed in both ATF4wt and ATF4ΔRK samples. Upregulated genes in ATF4wt and ATF4ΔRK are highlighted showing different pattern of expression. Color scale reflects expression level; black indicates no difference to control sample and green indicates increased expression compared to control. (b) Venn diagram depicting changes in genes of HL-1 cardiomyocytes overexpressing ATF4wt compared to HL-1 cardiomyocytes overexpressing ATF4ΔRK. Numbers of ATF4wt and ATF4ΔRK binding genes, with at least a fold change ≥ 1.2 or ≤0.83, and *p* value ≤ 0.05 compared to cells overexpressing GFP is indicated in circles (upper panel). For the grey area (genes enriched in ATF4wt but not in ATF4ΔRK), a gene ontology analysis was performed (lower panel). The main terms and pathways enriched only in the ATF4wt binding sites dataset are shown along with corresponding *p* values. (c) Venn diagram depicting changes in ATF4 binding genes in ATF4wt and ATF4ΔRK overexpressing cardiomyocytes in comparison to genes that are enriched in the stimulated cardiomyocytes overexpressing ATF4wt versus nonstimulated cardiomyocytes overexpressing ATF4wt microarray gene expression analysis. Numbers of genes, with at least a fold change ≥ 1.2 or ≤0.83, and *p* value ≤ 0.05 compared to cells overexpressing GFP are indicated in circles (upper panel). For the grey area (genes enriched in ATF4wt ChIP and stimulated cardiomyocytes overexpressing ATF4wt gene expression array only), a gene ontology analysis was performed (lower panel). The main terms and pathways are shown along with corresponding *p* values.

Thus, ATF4 seems to favor the expression of a number of defined target genes that are associated with cell stress and stress adaptive pathways. These target genes may be either induced directly by binding of ATF4 to its promoters or subsequently as a consequence of ATF4 induced adaptive pathways. In order to identify ATF4's primary target genes that are regulated directly by binding of ATF4 to its promoters, ATF4 and its non-DNA-binding mutant (ATF4ΔRK) as well as GFP were overexpressed in HL-1 cardiomyocytes, followed by Chromatin Immunoprecipitation and then fed onto a “ChIP-on-chip” assay. ATF4wt and ATF4ΔRK overexpression led to a regulation of genes with a significantly different expression level (fold change ≥ 1.2 or ≤0.83) in ATF4wt and ATF4ΔRK overexpressing cardiomyocytes in comparison to the GFP dataset. These genes were contrasted in a heatmap (Figure 4(a)). Several genes were regulated by ATF4wt as well as by ATF4ΔRK. In total, 2015 genes were subject of regulation compared to GFP overexpressing cardiomyocytes (Venn diagram in Figure 4(b) (upper panel)). In order to identify genes that were specifically bound by ATF4 and to rule out nonspecific binding, 90 genes were identified that were bound by ATF4wt and did not appear in the ATF4ΔRK dataset (grey area in the Venn diagram of Figure 4(b)). In the pathway enrichment analysis, this group of genes can be attributed to molecular pathways of amino acid biosynthesis, endoplasmic reticulum stress, and cell death (Figure 4(b), lower panel). Trib3, Ddit3, and ATF3 belong to this group (Table 1(b)). Trib3 is a well characterized ATF4 target gene. The putative protein kinase binds to ATF4 and inhibits its transcriptional activation activity. Ddit3 plays an essential role in the response to a wide variety of cell stresses and has been shown to induce apoptosis in response to ER stress [26].

Next, we identified genes that possess an ATF4wt DNA-binding site (ChIP-on-chip assay) and are regulated in stimulated cardiomyocytes with ATF4wt overexpression compared to nonstimulated cardiomyocytes with ATF4wt overexpression (mRNA microarray). 30 analog genes were found. This group of genes is involved in the ER stress response and apoptosis (Figure 4(c), lower panel, and Table 1(c)). ATF3 and Ddit3 were unregulated in this group of genes.

**Figure 5:**
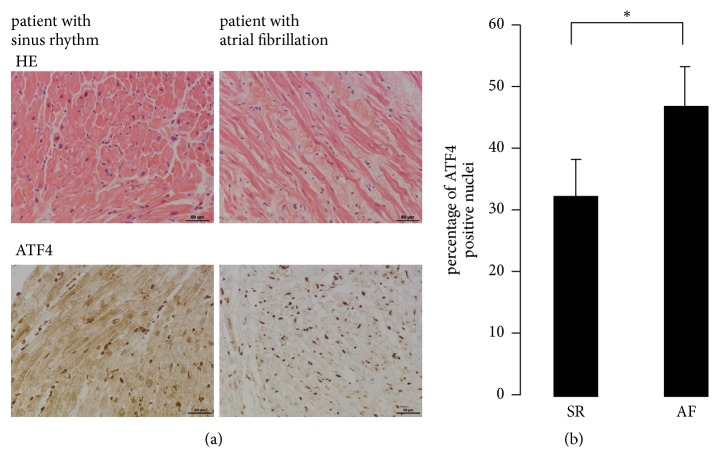
*Immunohistological analysis of ATF4 expression in atrial tissue obtained from patients with sinus rhythm and with atrial fibrillation*. (a) Representative images of HE staining and staining with antibodies directed against ATF4 (dark brown). Bar, 50 *μ*m. (b) Percentage of ATF4 positive nuclei in histological specimen obtained from patients in sinus rhythm compared to patients with atrial fibrillation. *∗* indicates *p* < 0.05.

In order to clarify ATF4's role in atrial fibrillation* in vivo*, the study was complemented with an immunohistological analysis of atrial tissue obtained from patients undergoing cardiac surgery (Table 2). Atrial tissue obtained from patients with a documented history of atrial fibrillation (*n* = 9) was compared to tissue obtained from patients without atrial fibrillation (*n* = 9). ATF4 could be detected with a nuclear localization predominantly in cardiomyocytes (Figure 5(a)). An elevated number of ATF4 positive cardiomyocytes was present in tissue specimen obtained from patients with atrial fibrillation compared to patients in sinus rhythm (Figure 5(b)); however, human ATF4 mRNA levels of tissue specimen detected by real-time PCR did not significantly differ between the groups.

**Figure 6:**
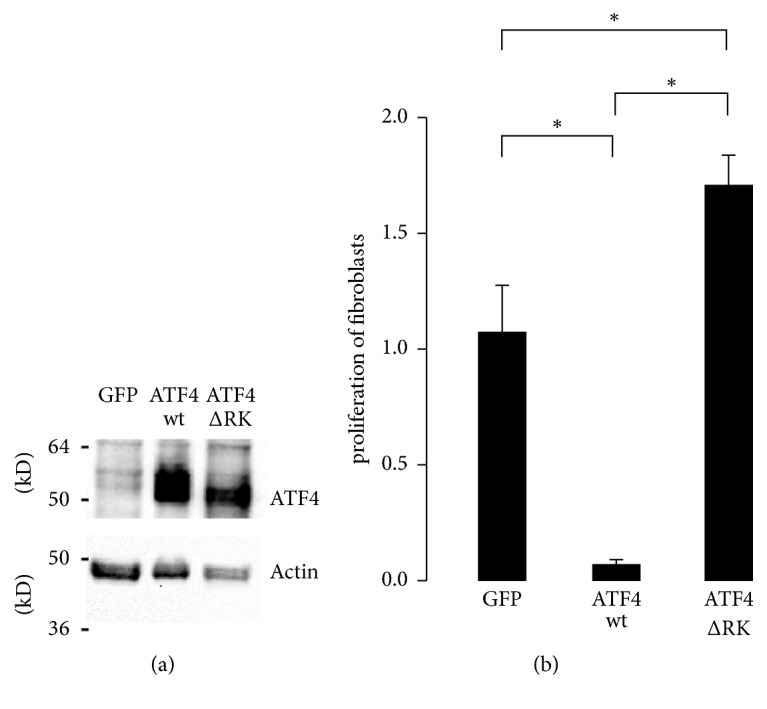
*ATF4 overexpression in fibroblasts*. (a) Representative western blots showing ATF4 expression in murine fibroblasts overexpressing GFP, ATF4wt, or ATF4ΔRK. (b) Cell proliferation was assessed by BrdU assay (*n* = 4). *∗* indicates *p* < 0.05.

In comparison to cardiomyocytes, cardiac fibroblasts did not display a strong ATF4 immunoreactivity. However, in order to assess the functional role of ATF4 expression in cardiac fibroblasts and in the development of fibrosis, we analyzed the effects of ATF4 overexpression in cultured cardiac fibroblasts. Fibroblasts were infected with GFP, ATF4wt, and ATF4ΔRK adenoviruses (Figure 6(a)). Subsequently, cell proliferation was assessed by a BrdU assay. The proliferation of murine fibroblasts was markedly reduced by ATF4wt overexpression compared to GFP overexpression. In ATF4ΔRK overexpressed cells, proliferation was increased compared to GFP overexpression (Figure 6(b)) suggesting a dominant negative effect on cardiac fibroblast proliferation.

## 4. Discussion

Solid evidence supports the hypothesis that atrial fibrillation is associated with both oxidative stress and endoplasmic reticulum stress in the atrial cardiomyocyte [27]. In fact, serum markers of oxidative stress have been shown to be elevated in AF patients [1]. Moreover, gene expression profiling in humans has revealed that AF is associated with a reduction of the expression of antioxidant genes and an increase in genes related to ROS suggesting that AF promotes a shift toward a prooxidant cell state in cardiomyocytes [8, 9]. Oxidants can modify ion channel activity and induce highly specific and tightly regulated cell stress signaling pathways that promote atrial remodeling. ER stress in atrial fibrillation is less well characterized. However, a recent report has demonstrated that tachypacing-induced apoptosis in atrial cardiomyocytes is regulated by ER stress-mediated MAP and MAPKs [10]. Correspondingly, chemical inhibitors of ER stress were shown to be partially protective suggesting that endoplasmic reticulum signaling is important for atrial cardiomyocyte apoptosis and remodeling in AF and a potential target for therapy. Moreover, ER stress could emerge from changes in glucose metabolism that would affect N-glycosylation in the ER and ER stress [28]. Indeed, diabetes seems to play an important role in the complex pathophysiology of atrial fibrillation.

Cardiomyocyte death is an important part of the atrial remodeling in response to atrial fibrillation. It is assumed that activation of specific cardiomyocyte prodeath transcription factors promotes the controlled demise of individual cardiomyocytes known as apoptosis. The current study provides insight into the transcription factors that regulate cardiomyocyte viability in cardiomyocytes that are exposed to atrial fibrillation. Specifically, we show that the transcription factor ATF4 is induced by both oxidative and ER stress. In consistence with the evidence that oxidative stress and ER stress are important components of the atrial cardiomyocyte remodeling process, we further demonstrate that AF induces ATF4 using a cellular model of atrial fibrillation. Moreover, forced expression of ATF4 sensitizes cardiomyocytes to cell death. Consistent with ATF4's role in regulating an upstream aspect of the cell stress induced death pathway, we found that ATF4 overexpression causes the induction of several apoptosis associated cell stress genes. Although known to be a stress-responsive protein, these results for the first time establish ATF4 as a protein that can be induced by tachypacing in cardiomyocytes and functions to lower the threshold for tachypacing induced death in cardiomyocytes.

ATF4 has an important role in regulating physiological responses to metabolic and redox processes and acts as a key transcription regulator of the integrated stress response (ISR) [6]. Classically, stress-mediated enhancement of ATF4 levels is known to occur via enhanced efficiency of translation of constant levels of ATF4 mRNA. Elevated levels of phosphorylated eukaryotic initiation translation factor 2 delay capacitation of reinitiating ribosomes, thereby fostering translation initiation at the ATF4 coding sequence, which subsequently permits ATF4 protein expression [11, 25]. Consistent with this model, we found an upregulation of ATF4 protein expression in tachypaced cardiomyocytes and cardiomyocytes exposed to ER stress and oxidative stress. In addition, we also observed an increase in ATF4 mRNA levels, thereby confirming reports describing a role for transcriptional regulation in ATF4 induction by different types of cell stress.

Originally described as a transcriptional repressor, ATF4 has been shown to have an activating effect on a number of several target genes, a significant part of which mediates cell stress responses and is involved in cell death and cell survival. Correspondingly, the gene array data presented in this study suggests that ATF4 acts at least in part as a transcriptional activator. Indeed, electrical stimulation in ATF4 overexpressing cardiomyocytes led to a pattern of gene expression characteristic of ER stress, oxidative stress, inflammation, and cell death suggesting that ATF4 can promote cardiomyocyte remodeling and cardiomyocyte death. Interestingly, ATF4 overexpression in unstimulated cardiomyocytes mainly influenced the expression of genes involved in amino acid biosynthesis suggesting that the effects of ATF4 expression are context specific.

Taken together, the data presented in this work are in support of a prodeath role of ATF4 in atrial cardiomyocytes. Generally, it is accepted that a short-lasting activation of the ISR is associated with an adaptive response in order to restore cellular homeostasis while a prolonged duration of ISR can cause the activation of prodeath pathways. ATF4 plays a key role in the switch between prodeath and prosurvival signaling by the ISR. In fact, previous findings regarding the roles of ATF4 in different tissues have yielded different results, and ATF4 is already known to have functions that are limited to a specific cell type. Specifically, ATF4^−/−^ fibroblasts are impaired in expressing genes involved in glutathione biosynthesis and resistance to oxidative stress [20]. However, ATF4 itself is capable of inducing cell death.* In vitro* studies in cortical neurons and an* in vivo* model of cerebral ischemia have yielded a prodeath role of ATF4 [29]. On the contrary, an elevated level of ATF4 expression has been shown in different cancer cell lines [30]. Different metabolic demands of individual cell types might help to explain the distinct role of ATF4 in cell death and survival. Moreover, differently expressed dimerization partners of ATF4 might also significantly impact its function. Interestingly, a recent study using mathematical modelling of the ISR has revealed that the ISR has three distinct activity states depending on the level and duration of stress. These activity states were associated with different outcomes with regard to cell death or survival [31]. Lamirault et al. [8] have carried out a detailed analysis of gene expression profiles associated with atrial fibrillation. Several of the genes that were regulated differently between patients in SR and AF include GATA4, glutathione peroxidase, and TNF. These genes have been linked to ER stress and ATF4 expression.

Histological analysis revealed a higher amount of ATF4 positive nuclei in AF patients compared to SR patients. However, the histological data must be interpreted with caution. Patients with mitral valve insufficiency are also at risk of AF even if in SR at time of surgery. The classification of sinus rhythm or atrial fibrillation was based on the available clinical data; however, documenting paroxysmal atrial fibrillation can be challenging in clinical practice. In fact, ATF4 mRNA levels did not differ significantly between the groups (data not shown). However, this finding does not necessarily argue against a prodeath role of ATF4 in atrial cardiomyocytes. ATF4 induced cell death might occur over a longer period of time, and ATF4 expression alone might not be sufficient to induce apoptosis in atrial cardiomyocytes* in vivo*. Moreover, surrounding fibroblasts and the intense chemical crosstalk between cardiomyocytes and cardiac fibroblast known to occur in cardiac tissue* in vivo* might also influence cell death/survival decisions in cardiomyocytes expressing ATF4. Overexpression of ATF4 in cardiac fibroblasts caused a marked inhibition of fibroblast proliferation; however, ATF4 expression in fibroblasts was low in human atrial tissue specimen. In addition, inflammation seems to be important for atrial fibrillation. A recent report has shown that type I IFN response could act as a potential therapeutic target for postmyocardial infarction cardioprotection [32]. On the contrary, our gene expression analysis demonstrates a downregulation of interferon genes by ATF4, indicating again that the effects of ATF4 seem to be both cell-type and context specific.

### 4.1. Limitations

The study has a number of important limitations. Atrial fibrillation is a complex arrhythmia, which critically depends on the presence of substrate and triggers, and therefore cannot be completely mimicked by single cell experiments. HL-1 cells have been abundantly used for* in vitro* studies, but a mouse cell line cannot reflect the complex behavior of human cardiomyocytes* in vivo*. In fact, future research is necessary to precisely characterize upstream effects and effector pathways of ATF4. However, our results demonstrate that rapid field stimulation leads to an induction of ATF4 mRNA and increased ATF4 expression on the protein level and decreased cell viability. Indeed, a direct translation to atrial fibrillation cannot be done on the basis of the data. On the contrary, the expression of ATF4 in human atrial cardiomyocytes suggests that ATF4 could be one of the transcription factors that are important for atrial cardiomyocyte survival and apoptosis in atrial fibrillation. Therefore, further studies will be necessary to precisely characterize ATF4's dimerization partners in cardiomyocytes and its downstream signaling events.

## 5. Conclusion

In atrial cardiomyocytes, ATF4 is expressed in response to oxidative stress and ER stress. Accordingly, rapid pacing of cardiomyocytes is associated with ATF4 induction. Forced expression of ATF4 reduces cardiomyocyte viability. Additionally, ATF4 is expressed in human atrial cardiomyocytes* in vivo*. Despite the inherent limitations of an* in vitro* model of AF, the results presented in this study therefore support the notion that ATF4 could play an important role in the atrial remodeling in atrial fibrillation.

## Figures and Tables

**(a):** 

Array ATF4wt stim versus GFP stim		
Gene Symbol	fold change	1/*p* value
Saa3	16.40	2.78E − 34
Avil	10.58	6.34E − 07
Gsta3	3.39	3.98E − 03
Gadd45a	3.22	7.62E − 22
Serpinf1	2.96	1.67E − 17
Csn3	2.69	2.78E − 34
Lcn2	2.69	4.88E − 04
Clec2e	2.59	1.67E − 17
Dgat2	2.51	1.43E − 07
Gulo	2.50	2.33E − 05
Hdc	2.44	1.73E − 02
Sprr2f	2.41	2.38E − 04
Colec11	2.40	3.51E − 03
Iyd	2.22	7.11E − 03
Iah1	2.21	2.07E − 05
Aqp9	2.17	1.29E − 06
Tg	2.13	2.49E − 02
Cox6a2	2.12	6.01E − 08
Was	2.11	2.86E − 05
Agpat9	2.08	4.97E − 03
Gp5	2.08	4.65E − 03
Reep6	2.07	1.93E − 03
ATF4	1.51	3.35E − 03
Ifit3	0.30	1.01E − 02
Irf7	0.38	1.57E − 07
Gbp3	0.40	1.31E − 02
Iigp2	0.43	2.06E − 07
Vwa5a	0.43	3.75E − 10
Igtp	0.43	2.71E − 02
Myadm	0.44	6.31E − 03
Lgals3bp	0.45	1.25E − 04
Parp14	0.45	8.69E − 03
Trim41	0.45	1.77E − 02
Galnt11	0.45	6.09E − 03
Batf2	0.47	4.82E − 06
Lgals9	0.49	1.72E − 02
Eif2ak2	0.50	4.89E − 04
Oas1b	0.50	3.76E − 03
Gbl	0.51	7.16E − 06
Irgb10	0.53	1.95E − 06
Tap1	0.53	3.22E − 02
Pln	0.53	1.46E − 05
Ifi35	0.54	7.86E − 06
Psmb9	0.54	4.91E − 04
Cxcl1	0.56	3.19E − 05
Wdr6	0.56	5.56E – 04

**(b):** 

ChIP ATF4wt w/o ATF4dRK	
Gene Symbol	−log⁡*p* value
Stk40	38.47
Ddit3	35.56
Arhgef33	35.47
BC071253	33.68
Herpud1	31.72
AK018753	29.82
Abcc8	27.51
AK140265	26.10
Gm13889	24.81
Gm10222	24.27
DQ539915	23.56
Rp9	22.95
Cox2	22.34
Atpase6	22.29
E2f4	21.63
Cytb	21.28
Atf3	20.76
Aars	20.29
Zxdc	20.02
Cebpb	17.44
Wnt3a	17.33
Rpn2	17.25
Atpase6	17.22
Ddr2	16.66
Siah2	16.34
Trim14	13.19
Apbb2	13.05
Eif1	11.93
Abhd11	11.57
Trmt12	11.27
Psat1	11.21
Pknox1	10.68
Med30	10.34
Abcc8	10.15
Mthfd2	10.08
Gars	10.04
Slc25a26	9.88
Nupr1	9.53
Nars	9.07
Fbln5	9.06
Trib3	8.14
Crip2	8.03
Asap1	7.97
Gm16197	7.93
Cebpg	7.81

**(c):** 

Array ATF4wt and ChIP ATF4wt		
Gene Symbol	fold change	1/*p* value
Asns	1.66	0.0002
Trib3	1.49	1.0000
Aars	1.38	0.0410
Psat1	1.37	1.0000
Aldh18a1	1.34	1.0000
Mthfd2	1.34	0.1265
Jdp2	1.33	0.8275
Eif2s2	1.31	0.1495
Rhbdd1	1.28	1.0000
Atad2	1.25	1.0000
Herpud1	1.23	1.0000
Nupr1	1.20	1.0000
S100a6	0.79	1.0000

**Table 2:** Patient characteristics.

Patient #	Rhythm	Age	Sex	Surgical treatment	CHD	CM	LA dilatation	aortic valve stenosis	mitral valve insufficiency
1	SR	58	m	aortic valve replacement and coronary bypass grafting				**X**	

2	SR	57	m	aortic valve replacement and coronary bypass grafting	**X**			**X**	

3	SR	56	m	acute mitral valve endocarditis; mitral valve replacement			**X**		**X**

4	SR	74	f	aortic valve and mitral valve replacement			**X**	**X**	**X**

5	SR	53	m	aortic valve replacement		**DCM**		**X**	

6	SR	74	f	coronary bypass grafting	**X**	**ICM**	**X**		

7	SR	78	m	aortic valve replacement				**X**	

8	SR	57	m	aortic valve replacement				**X**	

9	SR	79	m	aortic valve replacement and coronary bypass grafting	**X**			**X**	

10	AF	77	f	aortic valve replacement and coronary bypass grafting	**X**			**X**	

11	AF	81	m	aortic valve replacement			**X**	**X**	

12	AF	81	m	aortic valve replacement		**DCM**	**X**	**X**	

13	AF	58	m	mitral valve reconstruction			**X**		**X**

14	AF	73	m	aortic valve replacement and mitral valve reconstruction			**X**	**X**	**X**

15	AF	66	f	aortic valve replacement		**DCM**	**X**	**X**	

16	AF	68	m	aortic valve replacement and coronary bypass grafting	**X**	**ICM**	**X**	**X**	

17	AF	77	m	aortic valve replacement				**X**	

18	AF	75	m	aortic valve replacement and mitral valve and tricuspid valve reconstruction	**X**	**ICM**		**X**	**X**

List of 17 patients undergoing cardiac surgery (SR: sinus rhythm, AF: atrial fibrillation, CHD: coronary heart disease, CM: cardiomyopathy, DCM: dilated cardiomyopathy, ICM: ischemic cardiomyopathy, and LA: left atrium).

## Abbreviation

ATF4: Activating transcription factor 4
AF: Atrial fibrillation
bZIP: Basic leucine zipper
ER: Endoplasmic reticulum
MOI: Multiplicity of infection
ISR: Integrated stress response.

## Additional Points

*Highlights*

- Here, we demonstrate the expression of the bZIP transcription factor ATF4 in human atrial cardiomyocytes.
- In a cell culture based model of atrial fibrillation, ATF4 is induced by rapid pacing.
- Forced expression of ATF4 reduces cell viability.
- ATF4 binds to several promoters of cell stress genes and induces the expression of a number of ATF4 dependent cell stress genes.

## Supplementary Materials

Supplementary Materials Suppl. Figure S1: ATF4 expression in tunicamycin treated cardiomyocytes. Representative western blots showing protein expression of ATF4 in response to tunicamycin (0, 2, and 4 *µ*g/ml) treatment. Suppl. Figure S2: original western blots. (A, B) Blots from Figure 2(a). (C, D) Blots from Figure 1(b). (E, F) Blots from Figure 1(c). (G, H) Blots from Figure 1(d). (I, J) Blots from Figure 6(a) (K, L).
